# Cell-Penetrating Function of the Poly(ADP-Ribose) (PAR)-Binding Motif Derived from the PAR-Dependent E3 Ubiquitin Ligase Iduna

**DOI:** 10.3390/ijms19030779

**Published:** 2018-03-08

**Authors:** Ja-Hyun Koo, Heeseok Yoon, Won-Ju Kim, Donghun Cha, Je-Min Choi

**Affiliations:** 1 Department of Life Science, College of Natural Sciences, Hanyang University, Seoul 04763, Korea; koojh9036@gmail.com (J.-H.K.); geumbungeo@gmail.com (H.Y.); kwj8996@gmail.com (W.-J.K.); prankster119@daum.net (D.C.); 2 Research Institute for Natural Sciences, Hanyang University, Seoul 04763, Korea; 3 Center for Neuroscience Imaging Research (CNIR), Institute for Basic Science (IBS), Suwon 16419, Korea

**Keywords:** Iduna, cell-penetrating peptide, PAR binding motif

## Abstract

Iduna is a poly(ADP-ribose) (PAR)-dependent E3 ubiquitin ligase that regulates cellular responses such as proteasomal degradation and DNA repair upon interaction with its substrate. We identified a highly cationic region within the PAR-binding motif of Iduna; the region was similar among various species and showed amino acid sequence similarity with that of known cell-penetrating peptides (CPPs). We hypothesized that this Iduna-derived cationic sequence-rich peptide (Iduna) could penetrate the cell membrane and deliver macromolecules into cells. To test this hypothesis, we generated recombinant Iduna-conjugated enhanced green fluorescent protein (Iduna-EGFP) and its tandem-repeat form (d-Iduna-EGFP). Both Iduna-EGFP and d-Iduna-EGFP efficiently penetrated Jurkat cells, with the fluorescence signals increasing dose- and time-dependently. Tandem-repeats of Iduna and other CPPs enhanced intracellular protein delivery efficiency. The delivery mechanism involves lipid-raft-mediated endocytosis following heparan sulfate interaction; d-Iduna-EGFP was localized in the nucleus as well as the cytoplasm, and its residence time was much longer than that of other controls such as TAT and Hph-1. Moreover, following intravenous administration to C57/BL6 mice, d-Iduna-EGFP was efficiently taken up by various tissues, including the liver, spleen, and intestine suggesting that the cell-penetrating function of the human Iduna-derived peptide can be utilized for experimental and therapeutic delivery of macromolecules.

## 1. Introduction

Cell-penetrating peptide (CPP) is a short amino acid sequence that enables macromolecules such as proteins, DNA, and RNA to be transported into cells across the plasma membrane [1,2,3]. Until recently, the most widely used CPPs were TAT [4,5,6], Antp [7,8], VP22 [9], transportan [10], and poly-arginine [11,12]. Most CPPs, including the ones mentioned here, are derived from non-human sources, such as viruses or drosophila, or are synthesized artificially. Due to possible immunogenicity or toxicity of those CPPs, human-originated CPPs have been identified such as Hph-1 [13,14], Sim-2 [15], ECP^32–41^ [16] and Oct4 [17]. We also have previously reported novel human protein-derived CPPs including LPIN [18], 2pIL-1aNLS [19], dNP2 [20], and AP [21].

PolyADP-ribosylation (PARylation) is a post-translational modification that regulates various cellular processes, including DNA repair and cell death, via PAR polymerase [22,23,24]. Iduna (also known as RNF146) is a poly(ADP-ribose) (PAR)-dependent E3 ubiquitin ligase that can degrade PARylated proteins through PAR-dependent ubiquitination. In addition to proteasomal degradation, it regulates DNA repair, and cells overexpressing Iduna showed enhanced survival following γ-irradiation [25]. In vivo, Iduna protects against glutamate-induced brain tissue damage by inhibiting PAR polymer-induced cell death (parthanatos) [26]. The PAR-binding motif (F144 to R167) [27] of Iduna contains a highly cationic sequence, and in this study, we investigated the cell-penetrating ability of this sequence by generating recombinant proteins comprising the CPP conjugated with a fluorescent protein.

We hypothesized that this sequence from Iduna could be a novel human-derived CPP capable of transporting macromolecules across the plasma membrane. We cloned and purified a recombinant protein of the Iduna-conjugated enhanced green fluorescent protein (Iduna-EGFP) and tandem repeat form (d-Iduna-EGFP) to enhance delivery efficiency and nucleus localization. d-Iduna-EGFP showed robust delivery efficiency in Jurkat T cells and HeLa cells, and the efficiency was comparable to or better than that of TAT-EGFP. In the in vivo study in mice, d-Iduna-EGFP showed significant tissue localization efficiency, indicating the potential experimental or therapeutic application of the sequence for in vivo delivery of macromolecules into cells.

## 2. Results

### 2.1. CPP Candidate Sequence in Iduna Protein

**Figure 1:**
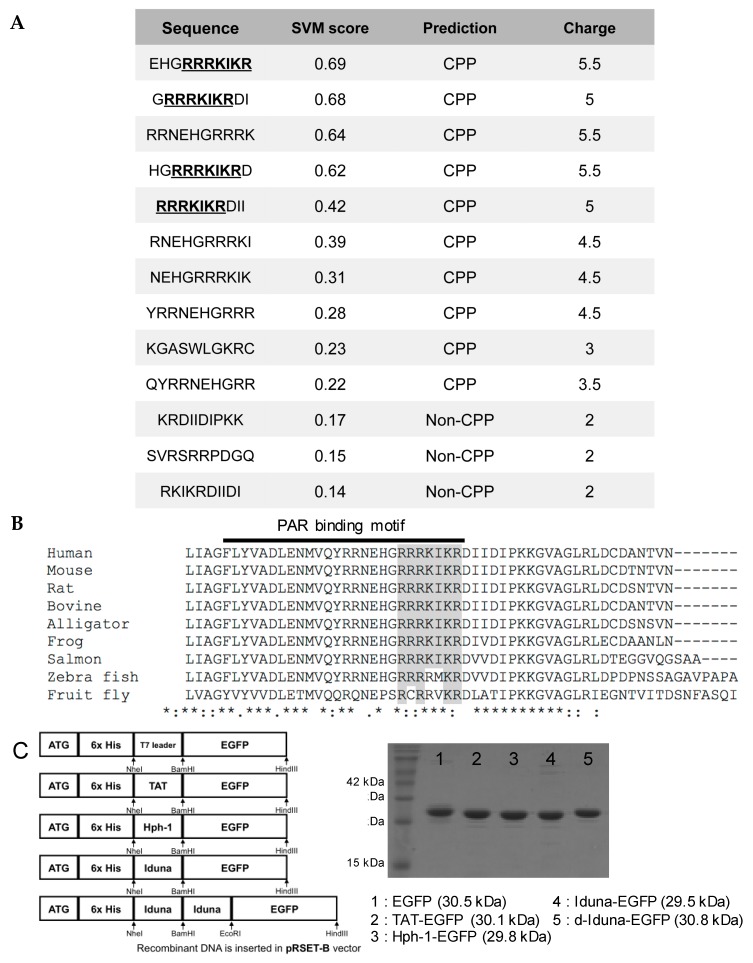
Identification of cell-penetrating peptide candidate in the PAR-binding motif of Iduna and generation of candidate sequence-conjugated recombinant protein. (**A**) In silico-based cell-penetrating peptide (CPP) prediction analysis (CellPPD) of the PBM sequence from Iduna. Candidate sequence is underlined; (**B**) Multiple alignment of candidate sequences from various species. Candidate sequence is highlighted. * fully conserved; : strongly similar properties; ∙ weakly similar properties; (**C**) Each DNA was cloned in pRSET-B vector; (**D**) SDS-PAGE analysis of purified recombinant proteins.

**Figure 2:**
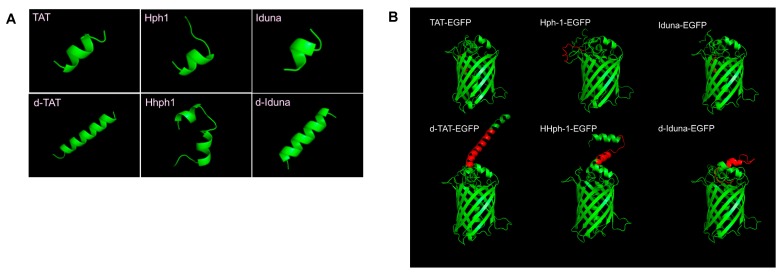
3D modeling of Iduna-derived sequence and the recombinant protein conjugated with EGFP. (**A**) 3D structure prediction of the Iduna-derived sequence or control CPP; (**B**) 3D structure prediction of the Iduna-derived sequence or control CPP (red) conjugated with EGFP (green).

To identify novel human-derived CPPs, we performed a motif scan analysis using the ScanProsite tool (available online: http://prosite.expasy.org/scanprosite/) of human proteins by filtering for R, K, I, L, V which are common amino acids in previously identified CPPs with a total length of 7 amino acids as minimum length. Among the candidates, we found the highly cationic sequence (RRRKIKR) in the PAR-binding motif (PBM) of Iduna. In addition, we utilized CPP prediction analysis (CellPPD, available online: http://crdd.osdd.net/raghava/cellppd/) [28] to confirm the CPP candidate sequence in Iduna. Four of the ten predicted CPP candidate sequences within Iduna that showed significantly high SVM scores (Support vector machine, possibility as an effective CPP) contained the RRRKIKR motif (Figure 1A). The PAR-binding motif (F143 to R168) of Iduna, including the candidate sequence, is highly conserved among various species. (Figure 1B). To test our hypothesis that the RRRKIKR motif from the PBM of Iduna can penetrate the plasma membrane and deliver a protein into the cell, we generated DNA constructs encoding a conjugate of the Iduna-derived sequence with EGFP (Iduna-EGFP), a tandem-repeat form (d-Iduna-EGFP), or controls, and cloned these in the pRSET-B vector (Figure 1C). The recombinants were expressed in an *Escherichia coli* BL21 (DE3) system and purified (Figure 1D) as previously described [19]. The 3D structure of CPPs was predicted using the modeling tool PEP-FOLD3 (available online: http://bioserv.rpbs.univ-paris-diderot.fr/services/PEP-FOLD3/). The modeling results suggesting both monomer and tandem repeat form of TAT, Hph-1, and Iduna form alpha helical structure (Figure 2A). The 3D structure of the EGFP-conjugated form was predicted using SparksX (available online: http://sparks-lab.org/yueyang/server/SPARKS-X/), and an extended alpha helical structure formed by the tandem-repeat sequences was predicted, along with the possible location and structural features of the recombinant proteins (Figure 2B).

### 2.2. Protein Delivery in Jurkat T Cells by the Iduna-Derived Sequence

**Figure 3:**
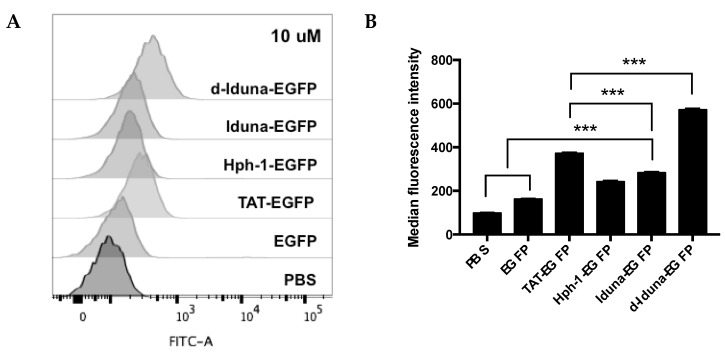

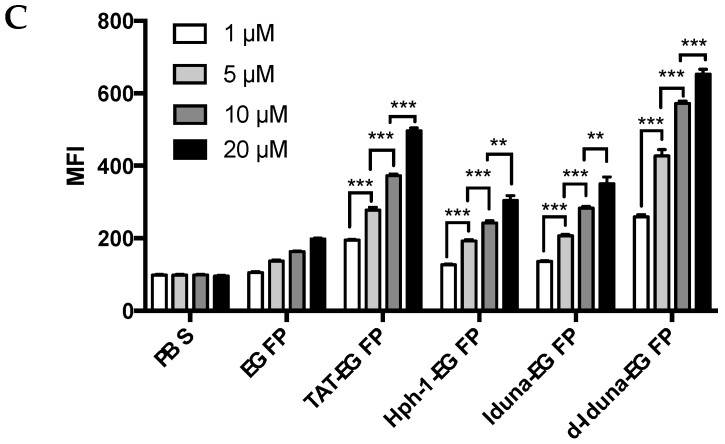
Comparative analysis of the protein delivery efficiency of the Iduna-derived sequence. (**A**,**B**) Jurkat cells were incubated with 10 μM Iduna-EGFP, d-Iduna-EGFP and other controls for 1 h at 37 °C. Intracellular fluorescence was analyzed by flow cytometry; (**C**) Jurkat cells were incubated with 0–20 μM Iduna-EGFP, d-Iduna-EGFP, and other control proteins for 1 h at 37 °C. For all experiments, cells were washed with PBS and trypsin to remove cell membrane-bound recombinant proteins. ** *p* < 0.005, *** *p* < 0.001, *n* = 3.

**Figure 4:**
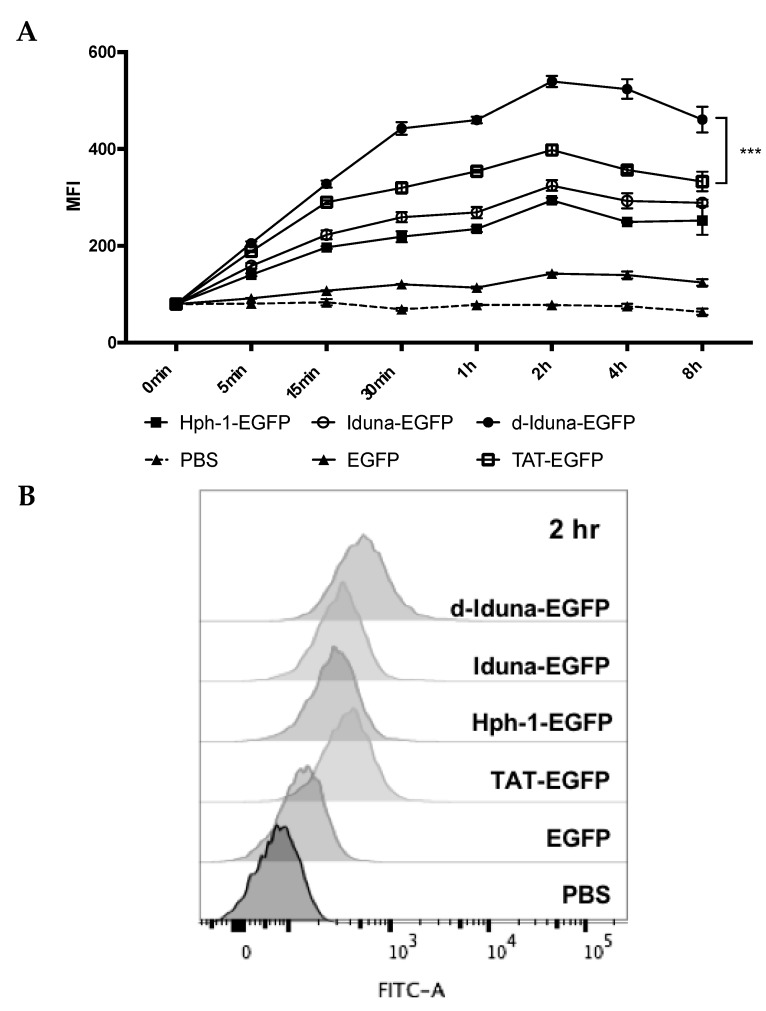

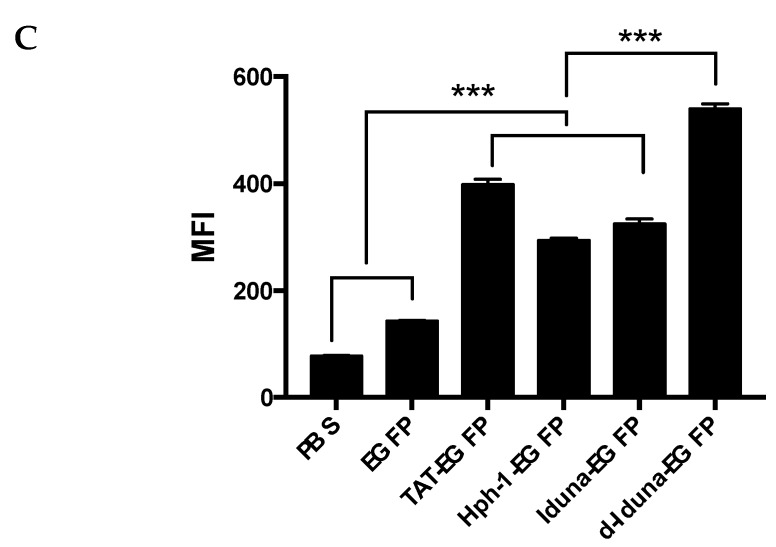
Time kinetics of the cell penetration efficiency of the Iduna-derived sequence. (**A**) Jurkat cells were incubated with 10 μM d-Iduna-EGFP and other control proteins for 0–8 h at 37 °C. Intracellular fluorescence was analyzed by flow cytometry; (**B**,**C**) Jurkat cells were incubated with 10 μM d-Iduna-EGFP and other control proteins for 2 h at 37 °C. For all experiments, cells were washed with PBS and trypsin to remove cell membrane-bound recombinant proteins. *** *p* < 0.001, *n* = 3.

To determine the protein delivery efficiency of the Iduna-derived sequence, we incubated Jurkat T cells with 1–20 µM Iduna-EGFP, d-Iduna-EGFP, or other controls for 1 h, and analyzed intracellular EGFP fluorescence by flow cytometry. EGFP uptake by cells treated with 10 µM Iduna-EGFP was two-times higher than that of PBS- or EGFP-treated control groups, and the protein delivery efficiency of Iduna-EGFP was comparable to that of Hph-1-EGFP (Figure 3A,B). Moreover, d-Iduna-EGFP showed 1.5-times higher protein delivery ability than its monomeric form or TAT-EGFP. These results suggested that the Iduna-derived cationic sequence is a CPP that can efficiently deliver a protein into cells and that the use of tandem repeats of the sequence significantly enhances the delivery efficiency. In addition, for all CPPs, the intracellular EGFP fluorescence increased dose-dependently (Figure 3C). Next, to determine the time kinetics of intracellular delivery of Iduna-EGFP, we incubated Jurkat T cells with 10 µM conjugates from 5 min up to 8 h and analyzed intracellular fluorescence by flow cytometry. After incubation for just 5 min, fluorescence of the d-Iduna-EGFP-treated group was significantly higher than that of the PBS- or EGFP-treated control groups, and the protein delivery efficiency increased gradually and time-dependently up to 2 h and then decreased slightly thereafter (Figure 4A). At 2 h, d-Iduna-EGFP-treated cells showed significantly higher intracellular fluorescence intensity than TAT-, Hph-1-, and Iduna-EGFP-treated cells (Figure 4B,C). These findings suggest that the Iduna-derived cationic peptide could deliver proteins into Jurkat T cells like other CPPs.

### 2.3. Intracellular Protein Delivery Mechanism of the Iduna-Derived Sequence

**Figure 5:**
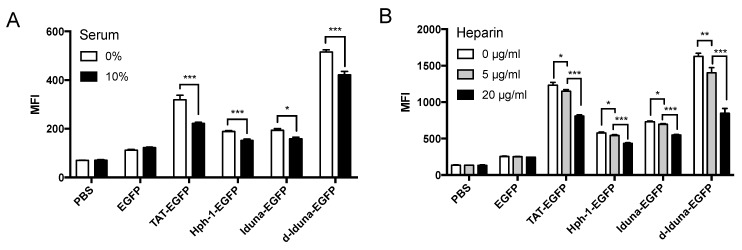

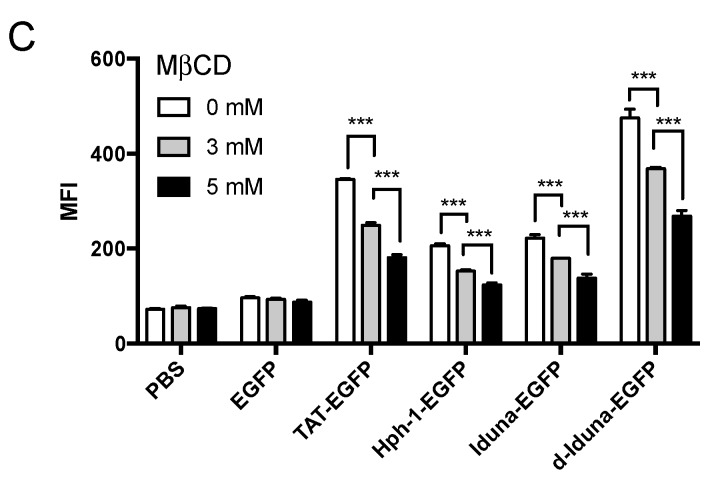
The Iduna derived sequence is internalized by cells via heparan sulfate, protein-protein interaction, and lipid-raft mediated endocytosis. (**A**) Jurkat cells were incubated with 10 μM d-Iduna-EGFP and other proteins for 1 h at 37 °C in 0% or 10% serum containing medium; (**B**) Jurkat cells were pre-incubated with 0–20 μg of Heparin or (**C**) 0–5 mM MβCD (methyl-β-cyclodextrin). After 30 min, 10 μM d-Iduna-EGFP and other proteins were added and incubated for another 1 h at 37 °C. Intracellular fluorescence was analyzed by flow cytometry. For all experiments, cells were washed with PBS and trypsin to remove cell membrane-bound recombinant proteins. * *p* < 0.05, ** *p* < 0.005, *** *p* < 0.001, *n* = 3.

To determine the cell-penetrating mechanism of Iduna-EGFP, we first incubated Jurkat T cells with d-Iduna-EGFP in serum-free or 10% serum-containing medium. Protein delivery by Iduna-EGFP and d-Iduna-EGFP was 20% less in the presence of 10% serum (Figure 5A), suggesting competition with other proteins for cell surface interaction sites. Next, we examined whether Iduna-EGFP and d-Iduna-EGFP interacted with heparan sulfate, a proteoglycan present in the plasma membrane and known to interact with CPPs [29,30,31]. We pre-incubated the cells with heparin (0–20 µg/mL) which is the soluble form of heparan sulfate for 30 min and treated recombinant proteins. The delivery efficiency of both Iduna-EGFP and d-Iduna-EGFP was 50% less in heparin-treated cells (Figure 5B), which was consistent with the results for TAT and Hph-1. This result suggested that both Iduna-EGFP and d-Iduna-EGFP interacted with proteoglycans on the cell surface. We further hypothesized that Iduna-EGFP and d-Iduna-EGFP utilize endocytic pathways, the most typical mechanism for CPP internalization [32,33,34]. To confirm our hypothesis, we treated the Jurkat T cells with 0–5 mM methyl-beta-cyclodextrin (MβCD), which can deplete cholesterol in the plasma membrane and inhibit lipid-raft-mediated endocytosis, during incubation with Iduna-EGFP and d-Iduna-EGFP. Intracellular delivery of Iduna-EGFP and d-Iduna-EGFP 40% decreased upon MβCD treatment (Figure 5C), suggesting that Iduna-EGFP and d-Iduna-EGFP were internalized via lipid-raft-mediated endocytosis. These results showed that, like other CPPs, the Iduna-derived sequence delivers proteins into Jurkat T cells through lipid-raft-mediated endocytosis following heparan sulfate interaction.

### 2.4. In Vitro Localization of the Iduna-Derived Sequence

**Figure 6:**
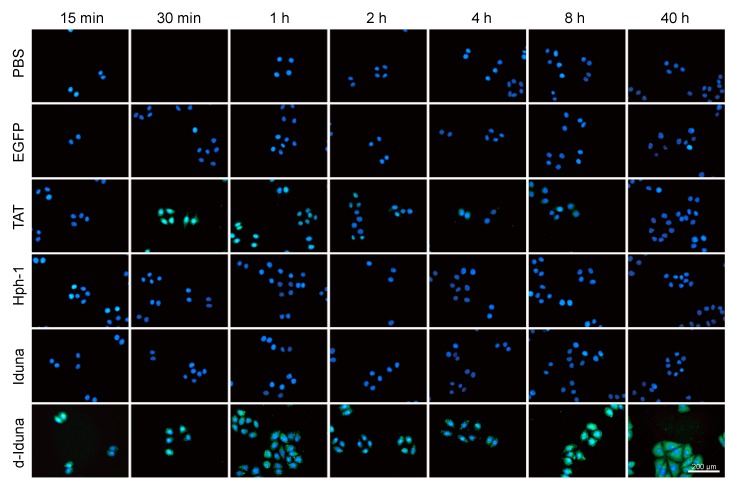
The Iduna-derived sequence localized to the cytosol and nucleus upon internalization by cells. HeLa cells were incubated with 20 μM d-Iduna-EGFP and other control proteins for the indicated time. After incubation, cells were washed twice with PBS, fixed with 4% paraformaldehyde, and nuclei were stained using Hoechst. Intracellular fluorescence was visualized by fluorescence microscopy at 200× magnification.

Due to the remarkable potential of the Iduna-derived sequence to deliver proteins into Jurkat T cells, we next examined the intracellular localization of the delivered proteins. For this, we incubated HeLa cells with 20 µM Iduna-EGFP and d-Iduna-EGFP for various durations (from 15 min to 40 h). After incubation, the cells were fixed and stained by Hoechst to visualize the nuclei. d-Iduna-EGFP-treated cells have the brightest green fluorescence in both cytoplasm and nucleus at all time points (Figure 6). Intracellular localization of TAT-EGFP was significant at 30 min and 1 h while the signal was hardly observed at 2–40 h. The signal of Hph-1 or Iduna-EGFP-treated cells was comparable with PBS- and EGFP-treated cells. Interestingly, although the TAT-EGFP signal in HeLa cells increased gradually up to 1 h and decreased thereafter, the d-Iduna-EGFP signal remained constant or increased up to 40 h. This result suggested that d-Iduna-EGFP offers sustained intracellular protein delivery as well as EGFP protein localization to the nucleus and the cytoplasm of HeLa cells.

### 2.5. In Vivo Tissue and Cellular Localization of d-Iduna-EGFP in Mice

**Figure 7:**
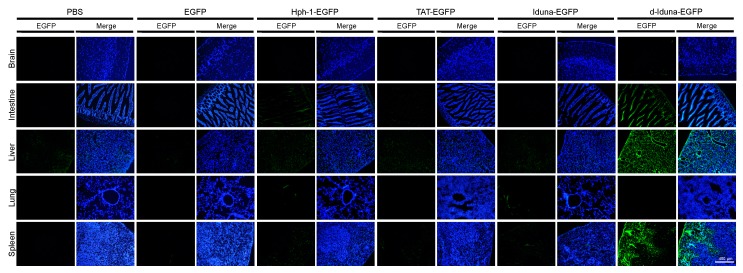
Localization of the Iduna-derived sequence in the intestine, liver, and spleen upon in vivo intravenous injection into mice. Six- to eight-week-old mice were intravenously administered 5 mg of d-Iduna-EGFP and other control proteins. Tissue fluorescence was analyzed 2 h after injection by fluorescence microscopy at 100× magnification.

Based on the superior in vitro performance of d-Iduna-EGFP compared with TAT-EGFP, we hypothesized that in vivo tissue or cellular localization of d-Iduna-EGFP could also be much better than that achieved using TAT-EGFP. To examine the in vivo localization of d-Iduna-EGFP, we intravenously injected 5 mg of d-Iduna-EGFP or other proteins into 6–8-week-old C57/BL6 mice. After 2 h, the tissues were harvested, fixed, and sectioned. The slides were stained with Hoechst and then analyzed by fluorescence microscopy (Figure 7). Significant tissue distribution of EGFP signal was detected in the liver, spleen, and intestine of the d-Iduna-EGFP-treated mice. Considering autofluorescence of the tissues in the fl-1 channel of the microscope and in vitro localization results, none of the other examined CPPs such as Hph-1, TAT-EGFP, and Iduna-EGFP were detected at significantly levels in the tissues. This finding suggested that d-Iduna-EGFP could deliver proteins into cells in vivo as well. From these results, we confirmed that the tandem-repeat form of the Iduna-derived sequence can be a novel CPP sequence for therapeutic protein delivery in vitro and in vivo.

## 3. Discussion

Iduna is PAR-dependent E3 ubiquitin ligase that regulates cellular responses such as proteasomal degradation and DNA repair [25]. These responses are mediated by the interaction between PAR and the PBM of Iduna. In this study, we found a highly cationic sequence in the PBM of Iduna and determined its cell penetrating function in vitro and in vivo.

A CPP is a short peptide that can penetrate the plasma membrane and deliver macromolecules such as proteins and nucleic acids into the cell [1,2,3]. CPPs have been used to deliver various therapeutic cargo into cells to regulate cellular responses [35]. However, most previously reported CPPs, including TAT [4,5,6], Antp [7,8], VP22 [9], transportan [10], and poly-arginine [11], have a non-human origin with potential toxicity or immunogenicity, which limits their use in human trial [36]. Due to these limitations, human-derived CPPs are required for therapeutic application of CPPs in humans. In our previous studies, we performed a TAT-homology search in human protein databases and identified novel human-derived CPPs, including LPIN from phosphatase LPIN3 [18], 2pIL-1αNLS from cytokine IL-1α [19], dNP2 from novel LZAP-binding protein [20], and AP from neuronal adhesion protein astrotactin 1 [21]. Here, we used the motif scan method, web-based approach, in combination with CPP candidate search to identify a novel human protein-derived CPP. We utilized the ScanProsite tool in UniProtKB and the CellPPD tool for in silico screening of human-derived CPPs.

The mechanism through which CPPs are internalized by cells has not been resolved, but various studies have reported that endocytosis via heparin sulfate interaction is the main route [29,30,31]. Consistent with these report, the Iduna-derived sequence was internalized via heparan sulfate interaction and lipid-raft mediated endocytosis. Our modeling analysis revealed that the Iduna-derived sequence forms an alpha helical structure, and d-Iduna was predicted to have a more stable and longer alpha helical structure than single Iduna or TAT, which was an optimal structure for molecular interaction on cell surface.

Previously, we found the cell-penetrating ability of the CPP was increased in the tandem-repeat form, which was consistent with the results for Hph-1 and HHph-1 and for NP2 and dNP2. Hhph-1 showed higher Foxp3 delivery into the nucleus compared with Hph-1 for converting CD4^+^ T cells to suppressor cells to inhibit autoimmune diseases in mice [37]. 2pIL-1αNLS is the tandem-repeat form of pIL-1αNLS, which is derived from the NLS of human IL-1a. 2pIL-1αNLS also showed enhanced nuclear localization compared with pIL-1αNLS [19]. More recently, dNP2 found to perform significantly better than NP2 in crossing the blood–brain barrier and delivering proteins to T cells in the central nervous system [20]. We also attempted to compare the delivery efficiency between single form (TAT, Hph-1 and Iduna) and tandem repeated form (d-TAT, HHph-1 and d-Iduna) of each CPP and the results showed that delivery efficiency of tandem-repeated double forms significantly enhanced intracellular protein delivery (Supplementary Figure S1A–C). Interestingly, TAT-EGFP signal was not detected in the cells of tissues in vivo while d-TAT-EGFP signal was significantly observed in spleen, liver, and intestine which is similar to that of d-Iduna-EGFP treated group (Supplementary Figure S2). Importantly, 100 μM of d-TAT-EGFP treatment into HeLa cells in vitro showed significant cytotoxicity with 35% reduced viability after 24 h incubation suggesting there is a significant toxicity by d-TAT. However, cell viability was not affected by equal amount of d-Iduna-EGFP treatment (Supplementary Figure S3). Our previous studies and present results for d-Iduna indicate that the tandem-repeat form of CPPs increase their protein delivery efficiency in vivo, and this could be a promising optimization strategy in CPP-based drug development and d-Iduna has an advantage of safety or stability compare to d-TAT.

PARylation is a post-translational modification that regulates cellular processes such as DNA repair, chromatin reorganization, and cell death [22,23,24]. PARP is the enzyme that catalyzes PARylation of a target region. When single-strand break (SSB) of DNA is detected by PARP, it catalyzes PARylation of the SSB region, which leads to the recruitment of DNA repair enzymes containing PBMs [38,39]. Iduna is a PAR-dependent E3 ubiquitin ligase that also possesses a PBM, and its enzyme activity is dependent on this motif [25]. In this study, we identified the cell penetrating function of a human Iduna-derived sequence and demonstrated its ability to deliver macromolecules into cells in vitro and in vivo. Because the Iduna-derived CPP is derived from the PBM and previous reports have suggested that lysine is critical for interaction of the PBM with PAR, our future work would involve determining whether the Iduna-derived CPP interacts directly with PAR after internalization into cells and inhibits the interaction of other PBM-containing proteins.

## 4. Materials and Methods

### 4.1. Recombinant Protein Purification

Iduna and other control CPP-conjugated EGFP were expressed and purified using a bacterial system. The gene encoding CPP-EGFP was cloned in pRSET-B plasmids, which were then used to transform *E. coli* BL21 (DE3) pLysS. Colonies of transformants were inoculated in 50 mL of Luria-Bertani (LB) medium and incubated at 37 °C with shaking at 200 rpm. After 6 h, the culture was transferred into 500 mL of LB medium and incubated for 1–2 h. When the optical density of the culture at 600 nm reached 0.3–0.5, recombinant proteins were induced by overnight incubation with 0.1 mM isopropyl-b-d-thiogalactopyranoside at 20 °C, with shaking at 150 rpm. After induction, the culture was centrifuged, and the pellet was suspended in native condition lysis buffer (50 mM NaH_2_PO_4_, 300 mM NaCl, 20 mM Imidazole, pH 8.0). The suspended pellets were sonicated to disrupt the bacterial membrane, and the soluble fraction was harvested by centrifugation and filtered with a 0.45 µm filter (Sartorious, Gottingen, Germany). 6His-tagged proteins were incubated with Ni-NTA agarose (Qiagen, Hilden, Germany) for 1 h on the rocker. The proteins were applied into Poly-prep column (Bio-Rad, Hercules, CA, USA) and washed with native condition wash buffer (50 mM NaH_2_PO_4_, 300 mM NaCl, 50 mM Imidazole, pH 8.0) and eluted with native condition elution buffer (50 mM NaH_2_PO_4_, 300 mM NaCl, 250 mM Imidazole, pH 8.0). Eluted proteins were desalted using a PD-10 Sephadex G-25 column (GE Healthcare, Little Chalfont, UK) and quantified with Bradford assay (Bio-Rad) [19].

### 4.2. 3D Structure Modeling

The 3D structures of the Iduna-derived peptide and control CPPs were analyzed by PEP-FOLD3 (Available online: http://bioserv.rpbs.univ-paris-diderot.fr/services/PEP-FOLD3/) and that of recombinant Iduna-derived sequences conjugated with EGFP was analyzed by SparksX (Available online: http://sparks-lab.org/yueyang/server/SPARKS-X/).

### 4.3. Cell Lines and Cell Culture

Jurkat (human leukemia) cells were purchased from the American Type Culture Collection (ATCC, Manassas, VA, USA) and maintained in RPMI 1640 medium (Corning, Corning, NY, USA). HeLa (human cervix epithelial carcinoma) cells and NIH/3T3 cells were purchased from ATCC and maintained in DMEM (Corning). Media were supplemented with 10% fetal bovine serum and 1% penicillin/streptomycin. The cells were cultured at 37 °C in a 5% CO_2_ incubator.

### 4.4. In Vitro Delivery Efficiency

Jurkat cells (2.5 × 10^5^ per well) were seeded into 96-well plates and incubated with CPP-EGFP at the indicated concentration for the time. All of the delivery experiments were tested in 10% fetal bovine serum and 1% penicillin/streptomycin containing complete medium. After incubation, cells were harvested and washed once with PBS. To remove cell membrane-bound recombinant proteins, the cells were trypsinized at 37 °C for 10 min. After trypsinization, the cells were washed again with PBS, and intracellular fluorescence was analyzed using fluorescence-activated cell sorting (FACS) Canto II flow cytometer. Data were analyzed using FlowJo software (ver 10.1 Tree Star, Inc., Ashland, OR, USA).

### 4.5. Mechanism of Internalization

In serum dependent experiment, Jurkat cells were incubated in 96-well plates in serum free or 10% contained medium with 10 µM of CPP-EGFP for 1 h. In MβCD or heparin experiment, Jurkat cells (2.5 × 10^5^ per well) were incubated in 96-well plates and pre-treated with MβCD (0–5 mM), heparin (0–20 µg) at 37 °C for 30 min. After the pre-incubation, CPP-EGFP was added to the medium and incubated for 1 h at 37 °C. All experiments were washed and trypsinized after incubation. Intracellular fluorescence was analyzed by flow cytometry as described above.

### 4.6. Intracellular Localization

HeLa cells were seeded at a density of 1 × 10^5^ per well on cover glass placed in 6-well plates and incubated at 37 °C for 24 h for attachment. Next, 20 µM of recombinant proteins were added and incubated for the indicated time. After incubation, the cells were washed two times with PBS and fixed with 4% paraformaldehyde for 10 min, and the nuclei were stained with 0.01% Hoechst 33342 in PBS for 10 min and cells were washed twice with PBS. Intracellular fluorescence was analyzed by fluorescence microscopy (Leica DMi8, Wetzlar, Germany).

### 4.7. Mice

We purchased 6–8 weeks old mice from Orient Bio (Seongnam, Korea) and housed them in a specific pathogen-free facility at Hanyang University. Next, 5 mg of CPP-EGFP was injected intravenously to the mice. After 2 h, the mice were sacrificed, and blood vessels were perfused with PBS to eliminate remained blood in tissues. The tissues were harvested, washed, and fixed by 4% Paraformaldehyde Fixed tissues were frozen in optimal cutting temperature compound. Frozen tissues were sliced with 8 µm thickness and nuclei were stained using 0.01% Hoechst 33342 in PBS for 10 min. Fluorescence in tissues was analyzed by fluorescence microscopy. All mouse experimental procedures were approved by the Animal Care and Use Committees of Hanyang University (2017-0149A, permission date (7 August 2017).

### 4.8. Cytotoxicity Assay

5 × 10^3^ HeLa cells were seeded on a 96-well plate and incubated 37 °C for 12 h. After attachment, 20, 50 and 100 µM of recombinant proteins were incubated at 37 °C in medium. After 24 h incubation, medium was removed and 10% cell counting kit-8 (CCK-8) reagent (Dojindo, Kumamoto, Japan) contained medium was added to each well. Cell viability was analyzed by measuring absorbance at 450 nm by microplate leader (iMark, Bio-Rad).

### 4.9. Statistics

The data were statistically analyzed using two-tailed Student’s *t*-test. *p*-Values less than 0.05 were considered statistically significant.

## Supplementary Materials

Supplementary materials can be found at http://www.mdpi.com/1422-0067/19/3/779/s1.
