# Supplementary material for: Cell-Penetrating Function of the Poly(ADP-Ribose) (PAR)-Binding Motif Derived from the PAR-Dependent E3 Ubiquitin Ligase Iduna
Source: Int J Mol Sci. 2018 Mar 8;19(3):779. doi: 10.3390/ijms19030779 (PMC5877640; doi:10.3390/ijms19030779)
Supplement: Supplementary file 1 [file ijms-19-00779-s001.pdf]

Supplementary

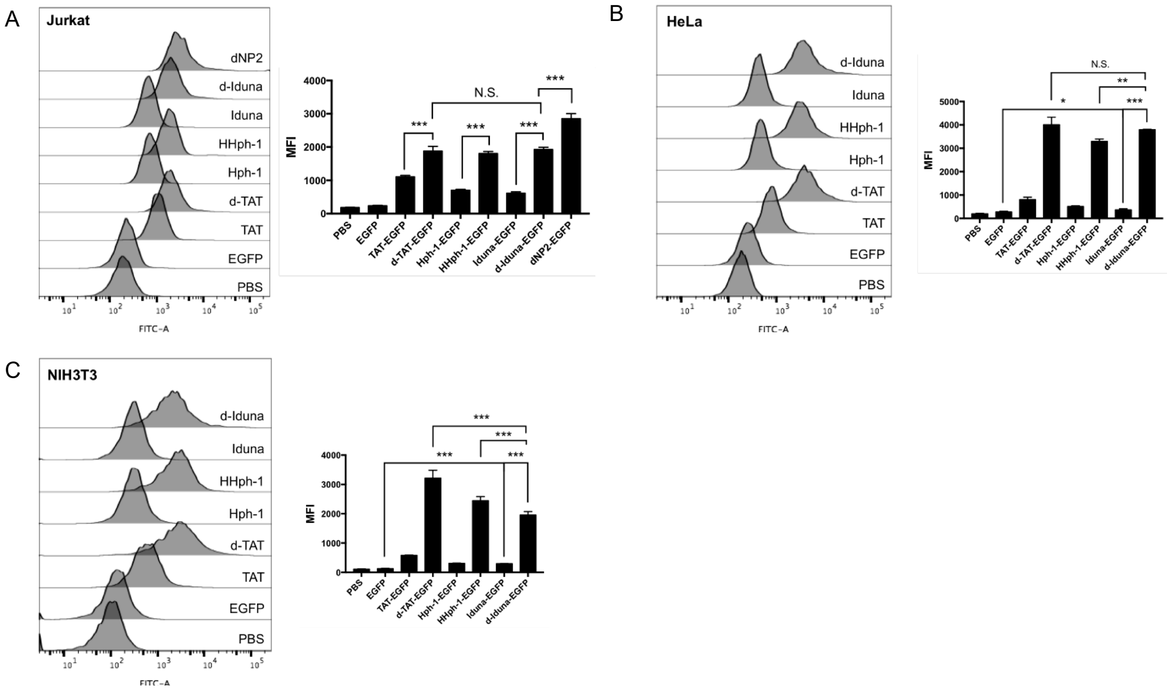

Figure S1. In vitro delivery efficiency of single form or tandem repeat form of Iduna derived sequence and other CPPs. Jurkat (**A**), HeLa (**B**) or NIH3T3 (**C**) cells were incubated with 10 μM of Iduna-EGFP, d-Iduna-EGFP and other controls for 1 hour at 37 °C. Intracellular fluorescence was analyzed by flow cytometry. * p <0.05, ** p <0.005, *** p <0.001, n = 3.

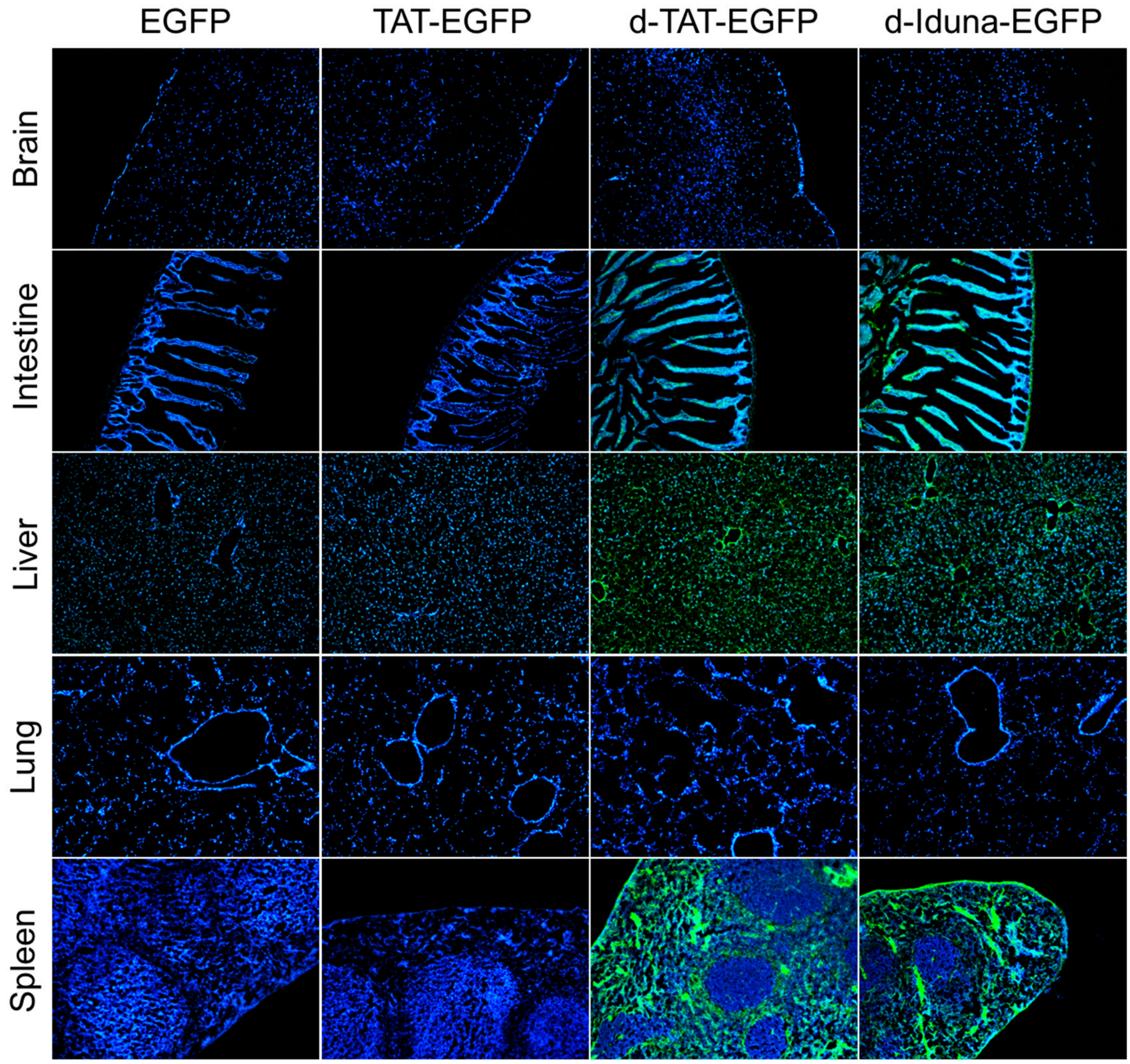

Figure S2. In vivo tissue distribution of d-Iduna-EGFP and d-TAT-EGFP upon intravenous injection into mice. 5 mg of d-Iduna-EGFP and other control proteins were injected into 6–8 weeks old mice intravenously for 2 h. Tissue fluorescence were analyzed by fluorescence microscopy with 100× magnification.

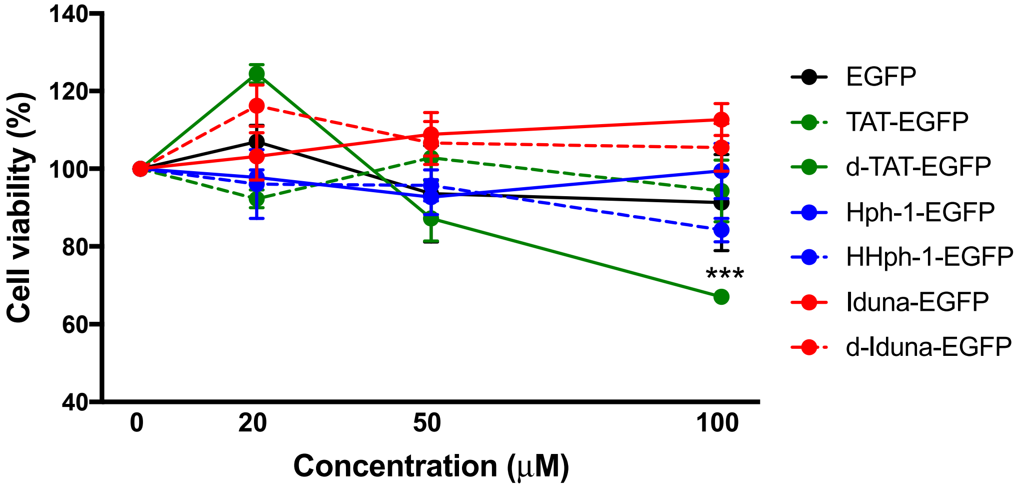

Figure S3. Cytotoxicity test of Iduna derived sequence. HeLa cells were incubated with 20, 50 and 100 μM of d-Iduna-EGFP and other control proteins for 24 h. The cell viability was measured by cell counting kit-8 (CCK8) assay. p-value of 100 μM of d-TAT-EGFP was compared with 100 μM of d-Iduna-EGFP. *** $p < 0.001$, $n = 3$.
